# Taurine and the renal system

**DOI:** 10.1186/1423-0127-17-S1-S4

**Published:** 2010-08-24

**Authors:** Russell W Chesney, Xiaobin Han, Andrea B Patters

**Affiliations:** 1 Department of Pediatrics, University of Tennessee Health Science Center, and the Children’s Foundation Research Center at Le Bonheur Children’s Medical Center, 50 N. Dunlap, Memphis, Tennessee, 38103, USA

## Abstract

Taurine participates in a number of different physiologic and biologic processes in the kidney, often reflected by urinary excretion patterns. The kidney is key to aspects of taurine body pool size and homeostasis. This review will examine the renal-taurine interactions relative to ion reabsorption; renal blood flow and renal vascular endothelial function; antioxidant properties, especially in the glomerulus; and the role of taurine in ischemia and reperfusion injury. In addition, taurine plays a role in the renal cell cycle and apoptosis, and functions as an osmolyte during the stress response. The role of the kidney in adaptation to variations in dietary taurine intake and the regulation of taurine body pool size are described. Finally, the protective function of taurine against several kidney diseases is reviewed.

## Introduction

The interactions between the kidney and taurine are many and varied. Taurine participates in several biologic processes in the kidney, and the kidney influences specific aspects of taurine homeostasis [1]. The numerous physiologic regulators of taurine handling by the kidney have been recently reviewed [2]. Thus, this review will focus on several aspects of renal function in relation to taurine and will cover large biologic themes. In addition, the role of taurine in the pathophysiology of kidney disease will be examined.

The physiochemical properties of the ß-amino acid taurine are probably responsible for some of its biologic characteristics. It is readily soluble in aqueous solutions. Taurine is not incorporated into protein, and can serve as an intracellular osmolyte. The taurine molecule acts as a zwitterion at physiologic pH and resides within the cell in millimolar quantities. Its accumulation within the cell requires active transport from the extracellular environment, where it is found in only micromolar quantities [3]. It has the lowest pK_1_ and pK_2_ of all amino acids. Some of these properties lead to the role of conjugation of bile acids [4] and uridine in tRNA [5].

### Ion reabsorption

**Figure 1:**
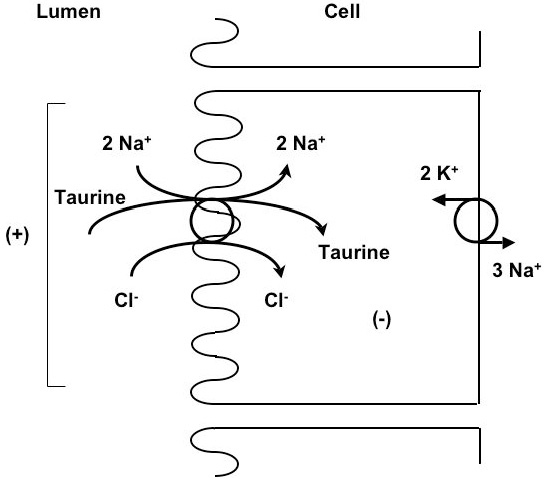
A model illustrates the 2 Na^+^:1 taurine:1 Cl^-^ stoichiometry of taurine transport.

**Figure 2:**
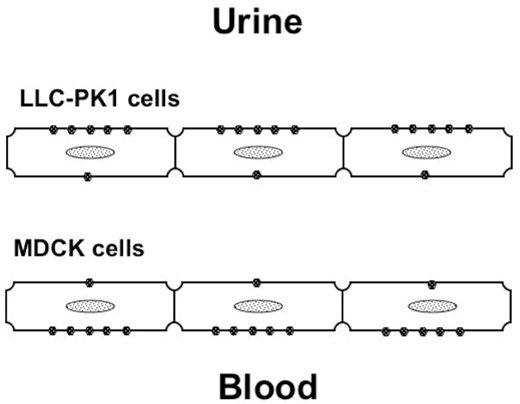
Taurine transport is membrane surface-specific.

The active uphill transport of taurine occurs via a sodium-dependent transporter (TauT) [6]. In addition to sodium, taurine uptake by renal epithelia requires chloride or bromide [7]. The model that best describes this transport is 2 Na^+^:1 taurine:1 Cl^-^ (Figure 1). Sodium and chloride move into cells by means of an external to internal downhill Na^+^ gradient (a chemical gradient), and then the sodium is pumped out of the cell by Na^+^K^+^-dependent ATPase. Taurine transport is stereospecific, inhibited by other ß-amino acids and GABA (gamma-aminobutyric acid) but not by α-amino acids, and is membrane surface-specific. In a proximal tubule cell line (LLC-PK1), uptake is maximal on the apical surface; in a distal tubule cell line (MDCK), uptake occurs at the basolateral surface (Figure 2) [8].

Taurine efflux from renal cells is dependent on the intracellular taurine concentration and requires the presence of both Na^+^ and Cl^-^ in the system. It does not contribute to the renal adaptive response described below. Efflux is much slower than uptake and has a higher K_m_. That taurine egress is dependent on specific ions suggests that it is not purely passive diffusion, but probably involves a carrier-facilitated process [9].

Taurine and its transporter also interact with glucose. Taurine in the glomerular ultrafiltrate appears to blunt the rate of Na^+^-dependent uptake of glucose by renal tubules and can potentially lead to glucosuria. While it is tempting to assume that taurine molecules in the tubular lumen compete for sodium and hence reduce glucose uptake, the much higher concentration of glucose (5.0 mM) makes this unlikely. Inhibition of the Na^+^-independent glucose transporter 1 (GLUT1) in activated macrophages (RAW264.7 cells) by taurine chloramine represents one mechanism by which inflammatory cell function can be modulated [10]. Some form of allosteric competition between taurine and GLUT1 may be relevant, but GLUT1 is commonly inhibited by vitamin C [11] rather than by amino acids. Also, because taurine is known to enhance insulin secretion [12], it may indirectly enhance glucose entry into cells. Hence, taurine may influence the intracellular as well as the transcellular movement of glucose.

### Renal blood flow

Taurine has several effects on renal blood flow and endothelial cell function. Sato *et al.* used the deoxycorticosterone acetate (DOCA)-salt rat model to study the various vasoconstrictive and vasodilatory properties of taurine [13]. Taurine status in the rat can influence renal vascular resistance [14-16], autonomic nervous control of arterial blood pressure [17,18] and the renal response to high sugar intake-induced baroreceptor reflex dysfunction [19]. Prenatal taurine exposure has long-term effects on arterial blood pressure and renal function in adult life.

Using the L-nitro-arginine methyl ester (L-NAME) hypertension model in the rat, Hu *et al.* have shown that taurine supplementation leads to increased serum levels of nitric oxide (NO) and NO synthase activity [14]. In addition, there is reduced renin-angiotensin-aldosterone axis activity and blunted elevation of cytokine and endothelin levels [14]. Taurine administration also ameliorates hypertension in hypertension-prone Kyoto rats [17].

Under certain circumstances, taurine depletion in fetal or perinatal rats results in higher blood pressure in adulthood [15,18-20]. Because of renal immaturity, and the extremely high fractional excretion of taurine, much of the taurine administered to rat pups is excreted in the urine [21]. Hypothetically, this taurinuria could result in volume depletion with a chronic up-regulation of the renin-angiotensin system (RAS) [22]. Whether this leads to imprinting and overactivity of the RAS is unclear.

Taurine has been examined as a renoprotective agent in several rat models [23-26]. The amino acid has been shown to be renoprotective in both healthy and diseased rats on salt- and fat-supplemented diets. If treated with enalapril (to block the RAS) or taurine, both hypertensive and glucose-intolerant rats will manifest a significant reduction in urinary protein excretion. In addition, rats fed high salt or high fat diets will excrete more taurine, as do rats fed a high glucose diet. This taurinuria may relate to competition for either sodium-dependent transport processes, energy, or both.

### Antioxidant properties

There exists extensive information regarding the antioxidant properties of taurine and its derivatives [27-30]. For this review, we will focus on studies relevant to the renal system.

Trachtman *et al.* showed that culturing renal mesangial cells in the presence of high glucose concentration resulted in build-up of advanced glycosylation products that could limit cell growth. Addition of the antioxidants taurine and vitamin E reversed the growth inhibition [29].

The major mechanism of antioxidation is the reaction of taurine with hypochlorous acid (HOCl) to form taurine chloramine. In several models of glomerular disease involving macrophage invasion there is increased intracellular activity of myeloperoxidase to yield HOCl arising from H_2_O_2_ present in renal tissue. These reactive oxygen species (ROS) can lead to DNA oxidation, protein nitration, and lipid peroxidation of renal cells [27,28,30]. Furthermore, oxidants arising from puromycin- or adriamycin-induced renal injury in rats are diminished following administration of 1% taurine in the drinking water [29]. Taurine has also been associated with reduction in oxidant levels in diabetic nephropathy [31].

### Ischemia/reperfusion injury

A model of renal injury that involves antioxidant injury in the renal vessels is the renal ischemia/reperfusion model. When rat kidney undergoes 60 min of ischemia followed by 90 min reperfusion, there is a substantial rise in serum creatinine and fall in renal ATP content. Prior intravenous administration of taurine at 40 mg/kg significantly reduces injury, as reflected by final serum creatinine levels much lower than in control rats that did not receive taurine [32]. No protection in terms of ATP content was found. In a saphenous vein model, ischemia reperfusion significantly reduced endothelial cell survival by increasing both apoptosis and necrosis [33]. These changes were accompanied by higher intracellular ROS and calcium ions and a reduction in endothelial nitric oxide synthase expression. Administration of taurine either prior to or following ischemia also attenuated epithelial cell apoptosis and necrosis.

The addition of taurine to University of Wisconsin (UW) solution was able to reduce tissue alterations during hypoxia and reoxygenation and permitted recovery of energy metabolism in LLC-PK1 cells [34]. However, it is in hepatic tissue that taurine supplementation of UW solution is more dramatic in tissue preservation [32,34,35].

The most important role for taurine in oxidant injury is probably the local and systemic scavenging of ROS. Taurine chloramine has been shown to serve as an oxidant reservoir, exhibiting delayed oxidant effects or acting at a distant site [36]. This phenomenon is particularly noteworthy in phagocytes, which are a source of taurine-related antioxidants [37] and are prevalent in an early phase of inflammation in the glomerulus and tubules [29].

### Cell cycle and apoptosis

Evidence has emerged that taurine and its transporter, the TauT protein, are important in the regulation of the cell cycle and apoptosis of kidney cells [38]. Taurine accumulates within the cells via active transport by TauT, and, hence, the quantity of transporter protein in the cell membrane determines intracellular β-amino acid concentration [1]. Cisplatin, a nephrotoxic chemotherapeutic agent, reduces taurine accumulation in renal cells through a p53-dependent process in LLC-PK1 cells [38]. In human embryonic kidney cells (293 cells), cisplatin up-regulates the proto-oncogene c-Jun. These variable responses to the anti-tumor agent can be shown by reporter assay and analysis, DNA binding, and Western blots of taurine transporter protein in cells. The functional *TauT* gene plays a modifying role in cisplatin-induced renal injury, and the transcription rate for *TauT* is regulated by p53 and c-Jun. The balance of such regulation determines the rate of synthesis of TauT protein, and thereby influences the fate of renal cells.

**Figure 3:**
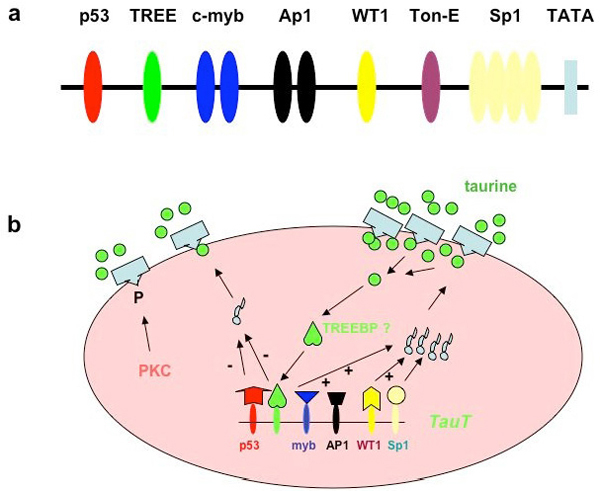
(a) The promoter region of *TauT* contains important binding sites. (b) Details of the intracellular signaling that regulate the gene.

The cell cycle-relevant pathway involving gene expression of cyclin-c and the *TauT* gene is cooperatively regulated by renal cells in response to hypertonicity [39] and reduced *TauT* promoter activity by doxorubicin-induced activation of p53. This p53 activation can be seen in human fetal kidney cells (293) and porcine proximal tubule cells (LLC-PK1), but in a cell line devoid of p53 expression, [10](1) cells, there is no repression of promoter [38]. With truncation of the *TauT* promoter or with mutation of the p53 binding site there is no repression of *TauT* activity. Activation of the *WT1* (Wilms tumor 1 gene) binding site in the promoter region up-regulates *TauT*, as does c-Jun. Figure 3 depicts the promoter region of *TauT* (3a) and the details of the intracellular signaling that regulate the gene (3b). Among the binding sites in the promoter region is a taurine response element (TREE) as well as the proto-oncogenes previously mentioned [1,2,38].

The product of *TauT* expression is TauT, a transporter protein containing 12 membrane-spanning domains inserted into the apical or basolateral membranes of renal cells. The taurine transporter has been cloned from several species and tissues, including rat brain [6] and dog kidney [40]. The genes encoding TauT in various species share a high degree of homology, residing on chromosome 6 in the mouse and 3p21-25 in man [41]. A renal adaptive response to taurine availability has been demonstrated in many mammalian species, including humans [42]. The mechanisms for this adaptive response, described in detail below, occur at the levels of transcription, translation, and post-translational modification [2]. Phosphorylation of serine 322 by protein kinase C (PKC) results in reduced transporter activity. This phosphorylation site is on the fourth intracellular loop (S_4_), a highly conserved motif in all mammalian species examined [6,40].

### Stress response and taurine as a renal osmolyte

**Figure 4:**
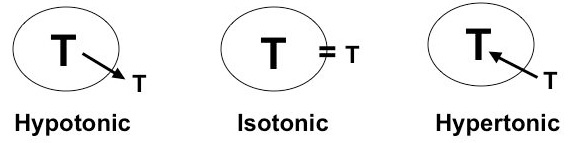
Taurine’s role as an osmolyte is shown by its net movement under different conditions of tonicity.

Sorbitol, myo-inositol, betaine, α-glycerophosphorylcholine and taurine have been identified as major osmolytes in the renal medulla [43-45]. The taurine uptake process responds to osmolar signals under three special circumstances: 1) In fish adapting from fresh water to sea water or vice versa [42,46,47]; 2) In the mammalian brain under conditions of hyper- or hyponatremia [48,49]; 3) In the unique osmolar environment of the renal medulla [43-45,50]. Osmolar regulation results in movement of taurine into or out of the medullary cell rather than transcellular movement (reabsorption) (Figure 4). The renal medulla is the site of urinary concentration or dilution, the countercurrent multiplier mechanism, and aquaporin activity to form water channels. It can establish an osmolar gradient of 50 to 1200 mOsm in man, and even steeper gradients in rodents [51]. Osmoregulation of taurine transport occurs in cells of the loop of Henle and the medullary collecting duct. The relevant biologic process is termed “cell volume regulation” [44,45,52], (Figure 4). Several studies have demonstrated that medullary cells in culture (MDCK or M1 cells) exhibit taurine transport across the basolateral surface rather than the apical surface [44,45,50]. A response to hyperosmolarity is not evident in proximal cell lines [50].

**Figure 5:**
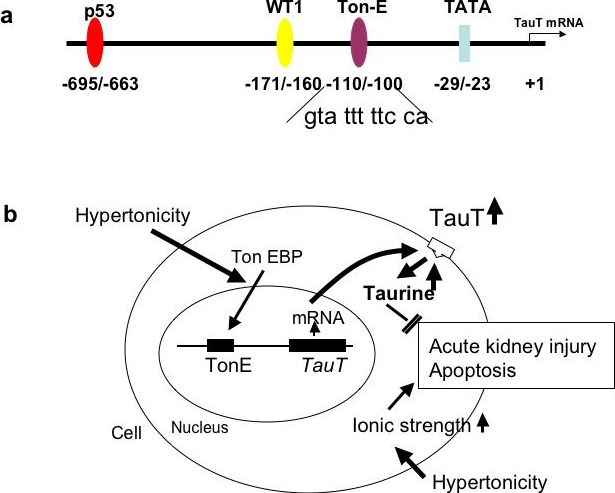
(a) Location of the TonE site on the *TauT* gene promoter region; (b) Model of TonE and *TauT* gene activity following exposure to hypertonic conditions.

Handler and Kwon have shown that cells that respond to hyperosmolar stress have a tonicity response element (TonE) that responds to a TonE binding protein (TonEBP) [44,53]. Extracellular sucrose or raffinose leads to increased binding of TonEBP to TonE, up-regulation of the genes for osmolar transporters (sorbitol, myoinositol, etc.), increased production of mRNA for TauT protein synthesis, export and insertion of protein into the basolateral cell membrane, and enhanced transport of taurine into the cell (Figure 5a) [44,50]. Ito *et al.* have recently shown that the TonE site is located on the promoter region proximal to -124 and distal to -99 (Figure 5b) [53]. A mutant TonE was unresponsive to hypertonicity. This study also demonstrates how the TonE/TonEBP system regulates cell volume and prevents hyperosmolar stress [53].

### Renal regulation of taurine body pool size

Humans and other mammals on diets of differing taurine or sulfur amino acid content excrete different amounts of taurine, particularly as a percent of the filtered load [2]. This is unusual because amino acid reabsorption by the kidney is highly efficient and generally exceeds 99%. Urinary taurine excretion is low when dietary taurine is restricted, as in a vegetarian diet. Conversely, when taurine intake is high, as in a diet rich in meat and seafood, taurine excretion is high. Han *et al.* review many of the specifics of this phenomenon [2].

Although renal regulation of ion reabsorption is a long-recognized concept in transport physiology, application of this principle to an amino acid is recent. Examined in terms of the fractional excretion of taurine, a variation of 0.5% to 80% has been found [54]. From a renal physiologic viewpoint, both an increase and reduction of urinary excretion suggest an adaptive regulation of transport, as is observed for the phosphate ion. We use the term “renal adaptive response to alterations in taurine intake” to describe these observations. Adaptation of the taurine transporter system is a limited phenomenon exhibited by the kidney and the gut, and under conditions of malnutrition [2]. From a nutritional perspective, all mammals should retain amino acids. However, because taurine is a β-amino acid and is devoid of a carboxyl group, it cannot be incorporated into protein and resides freely in intracellular water. Among other features is that taurine is not metabolized by eukaryotes and does not contribute to gluconeogenesis, but it does participate in conjugation of certain compounds (such as bile acids). It is largely inert and not a source of energy. These ideal physiochemical properties of taurine lead to a central hypothesis that taurine can be responsible for cell volume regulation, because taurine movement across the membrane surface of a cell “can evoke changes in the concentration of solutes and solvents within a cell” [52].

If taurine movement is important in the maintenance of cell volume, what regulates the transport from a dietary perspective? The transport of taurine *in vivo* appears to be precisely regulated by the kidney, and is mimicked *in vitro* in a variety of renal systems, including uptake into renal slices, renal cells in culture, isolated renal tubules, and isolated brush border membrane vesicles. It is regulated at both the level of mRNA transcription and protein synthesis [2].

The renal adaptive response was first described in rats fed a low taurine diet (LTD, containing suboptimal concentration of the precursor methionine), a normal taurine diet (NTD), or a diet supplemented with 3% (high) taurine (HTD) [54]. Specific taurine transporter mRNA levels are higher in LTD-fed rats and lower in HTD-fed rats as compared to NTD-fed rats. Western blot analysis shows more taurine transporter protein in membranes from LTD-fed animals and less in those fed HTD. The transcription rate is higher in cells in culture deprived of taurine, and lower in cells exposed to excess taurine [55-57]. Exposure of cells to β-alanine, which depletes intracellular taurine, leads to enhanced uptake. Likewise, *in vivo*, fasted rats show higher taurine reabsorption rates and increased uptake by brush border membrane vesicles [58]. Renal brush border membrane vesicles prepared from kidneys of taurine-deprived felines, who require dietary taurine to maintain usual tissue levels, show greatly enhanced taurine uptake [59]. This evidence indicates that whatever reduces intracellular taurine content up-regulates the *TauT* gene and synthesis of TauT protein. Likewise, with increased taurine availability, increased dietary intake and increased intracellular taurine concentration, the uptake of taurine by vesicles and cells is reduced and the process is down-regulated.

In an effort to clarify the signal for the up- or down-regulation, truncation analysis of the promoter region revealed that the taurine response element (TREE) resides between the c-myb and p53 binding sites (Figure 3a). Truncation proximal to this site blocks the adaptive response, as shown by reporter assay [2]. The molecule that TREE responds to is not established, but it is possible that it is the intracellular concentration of the taurine molecule per se.

**Figure 6:**
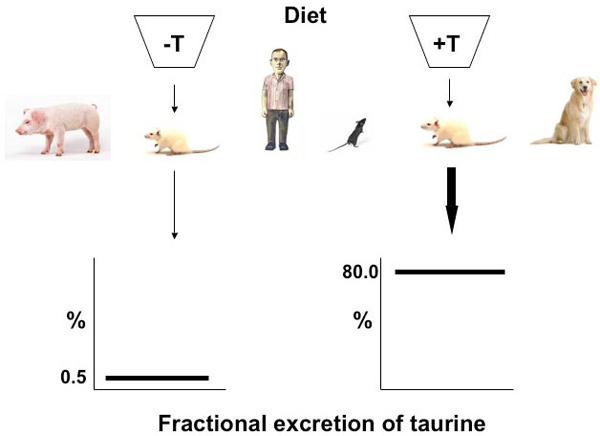
The renal adaptive response to dietary taurine intake conserves the total taurine body pool by reabsorbing or excreting taurine depending on its availability.

Plasma taurine levels do not vary greatly with the availability of dietary taurine. Using specific antibodies, taurine can be found in the nucleus, and thus is present at the site of transcription. Addition of taurine to cell cultures that have adapted to a low taurine environment can rapidly (within 8 hr) reverse the up-regulation response [8]. Both the rapid and the slower classic adaptive responses are found in numerous mammalian species, including man, dog, pig and rodent. It is evident in herbivores, carnivores and omnivores [2]. Depending on taurine intake, the urinary fractional excretion of taurine can vary from 0.5% to 80.0% (Figure 6).

### The role of taurine in the pathophysiology of the kidney

Taurine has been shown to play a role in four different forms of kidney disease: glomerulonephritis, diabetic nephropathy, chronic renal failure, and acute kidney injury (AKI). Much of the work on the role of taurine in relation to kidney disease has been performed in animal models, especially murine species. Many studies were performed nearly two decades ago and are descriptive, with the exception of the studies involving taurine chloramine. Only in the area of protection of the kidney against AKI have intracellular and molecular mechanisms been explored with the use of transgenic and knockout mouse models and knockdown cell lines.

### Protection against glomerulonephritis

Trachtman has reviewed the evidence that taurine functions as a protective agent against immune- or toxicity-induced forms of glomerulonephritis [29]. In the Masugi glomerulonephritis model, rat kidney homogenates are injected into rabbits. After several weeks, rabbit serum is injected into rats. There occurs a heterologous phase in which injected antibodies lead to the migration of neutrophils into rat glomeruli. Myeloperoxidase (MPO) located in neutrophils causes generation of radicals, including hypochlorous acid [28,30,60,61]. Hypochlorous acid activates tyrosine phosphorylation signal pathways, leading to calcium signaling and tumor necrosis factor α (TNFα) production [61]. In MPO^-/-^ mice, fewer reactants are generated [60].

Subsequently, in an autologous phase, T cells and macrophages invade. The addition of taurine chloramine to the diet appears to inhibit the function of antigen-presenting cells and T cells in T cell-induced crescentic glomerulonephritis [60]. Lian *et al.* showed that taurine in drinking water reduced urinary protein excretion, and both serum and urine platelet-activating factor (PAF) levels [62]. Renal cortex and medulla PAF values are also lower than in control rats.

Another component of glomerulonephritis is an increase in glomerular albumin permeability (GAP). In a model using isolated rat glomeruli, which are infiltrated by neutrophils, H_2_O_2_ alone does not increase GAP, but H_2_O_2_ and MPO together do increase GAP [63]. This increase can be inhibited by superoxide dismutase, catalase or taurine.

A model of chronic puromycin aminonucleoside nephropathy that resembles human focal segmental glomerulosclerosis (FSGS) can be induced in rats. When rats are given 1% (w/v) taurine in their drinking water, urinary albumin excretion, segmental glomerulosclerosis and tubulointerstitial injury are significantly diminished. The urine albumin/creatinine ratio is lower in taurine-supplemented animals, as are levels of the oxidant malondialdehyde in renal cortex. While the presumed mechanism of nephroprotection is the formation of taurine chloramine from taurine, this was not directly measured [64].

### Protection against diabetic nephropathy

Taurine has afforded renal protection against models of diabetic nephropathy [31]. The importance of this observation relates to the fact that diabetes mellitus (type 1 and type 2) is the predominant cause of end stage renal disease and the need for dialysis in North America [65]. In rats with streptozocin-induced diabetic nephropathy, addition of taurine to the drinking water and exogenous insulin inhibited the increase in glomerular planar area and ameliorated the condition, as did vitamin E [31]. Administration of vitamin E and taurine is associated with a reduction in advanced glycosylation end products and the extent of lipid peroxidation. Taurine can also neutralize the aldehydes of glycation end products. The formation of Schiff’s base between taurine and the aldehydes may diminish glucose toxicity. Taurine and its congeners reduce the formation of intracellular oxidants and afford protection against erythrocyte membrane damage [66], which could also reduce the fragility of erythrocytes within glomerular capillaries.

Another hypothesis concerning the importance of taurine in diabetic nephropathy involves the increased production of sorbitol. Simply stated, the elevated extracellular concentration of glucose disturbs cellular osmoregulation and sorbitol is synthesized intracellularly via the polyol pathway [67]. Intracellular accumulation of sorbitol crowds out other intracellular osmolytes, including taurine and myo-inositol. This disturbance of cell volume regulation might be altered by taurine supplementation, but this has not been tested [67].

### Protection against chronic renal failure

In general, human patients with chronic renal failure have reduced plasma and muscle intracellular concentrations of taurine [68]. However, an open label, non-randomized trial of taurine supplementation (100 mg/kg/day) in 10 hemodialysis patients resulted in extremely high taurine levels in plasma and muscle [69]. The plasma concentration rose from 50 μM to 712 – 2481 μM after 10 weeks of therapy, and muscle values more than doubled [69], likely because no renal adaptive response is possible in these patients and taurine cannot be excreted. Clearance by dialysis was not measured.

### Protection against acute kidney injury

Several models of AKI have been used to examine the influence of taurine in this process. In a gentamicin toxicity model, rats are injected with the aminoglycoside antiobiotic, leading to a rise in serum creatinine and histologic features of acute tubular necrosis. Administration of taurine attenuated the rise in creatinine and there was less accumulation of gentamicin [70]. In this model, the content of glutathione peroxidase and superoxide dismutase are similar in kidneys of taurine-treated rats and controls.

Acute kidney injury is a major problem in patients with sepsis, toxic injury and shock. The overall mortality rate is approximately 50% [71]. In cancer patients receiving chemotherapeutic agents, evidence of kidney injury, as defined by elevation of biomarkers, is common. Cisplatin is a frequently used chemotherapeutic agent, limited mainly by its nephrotoxicity. As many as 25% to 35% of patients experience a significant decline in renal function after a single dose of cisplatin [72].

Elevated expression of the tumor suppressor gene p53 has been detected in the kidneys of rats with cisplatin-induced AKI [73]. Jiang *et al.* have shown that p53 is an early signal in cisplatin-induced apoptosis in renal tubular cells [74]. These findings suggest that altered expression of distinct p53 target genes may be responsible for p53-induced progressive renal failure.

Our studies have shown that *TauT* is negatively regulated by p53 in renal cells [75]. Cisplatin, which stimulates p53 production, accumulates in all cell types of the nephron but is preferentially taken up by highly susceptible cells in the S3 segment of the proximal tubule [76], which is also the site where adaptive regulation of *TauT* occurs [77]. Cisplatin has been shown to impair the function of the taurine transporter and to down-regulate expression of *TauT* at the transcriptional level in a dose-dependent fashion [78]. We hypothesized that *TauT* plays a role as an anti-apoptotic gene and functions to protect renal cells from cisplatin-induced nephrotoxicity *in vivo*.

Transgenic mice over-expressing human *TauT* and wild-type mice were injected with cisplatin or saline; renal failure biomarkers (blood urea nitrogen, creatinine, urinary protein excretion) were measured and the mortality rate recorded [78]. Over-expression of *TauT* in the transgenic mice conferred significant protection against renal damage and death caused by cisplatin as compared to drug-treated control animals. Histological analysis of kidneys from cisplatin-treated transgenic mice showed greater amounts of membrane-bound TauT protein, higher levels of intracellular taurine, and less necrosis and apoptosis than the kidneys of cisplatin-treated control mice. The histological findings were similar to those found in saline-injected control animals [38].

### Physiologic roles for taurine relative to the kidney

It is possible to develop a structural-functional map of the kidney based upon information presented in this review. The nephron, the basic unit of the kidney, has several different cell types that behave in a variety of ways when interacting with taurine. The major characteristics of taurine in terms of kidney function are shown in Table 1. Although many of these roles may overlap in different renal tissue types, the function of each structural part sets the paradigm within which taurine will operate.

**Table 1:** The role of taurine in various renal structures

Renal Structure	Role of Taurine
Vasculature	Regulate blood flow
Glomerulus	Scavenge ROS (reactive oxygen species)
Proximal tubule	Na^+^ transport Regulate taurine body pool size
Medulla	Osmoregulation Cell volume regulation

The effect of taurine on renal blood vessels is to alter blood flow, and probably to stabilize the endothelium of the extensive renal vascular network [33]. Taurine influences blood flow within all types of vessels (capillaries, venules and arterioles) through several mechanisms discussed previously, such as NO synthase activity, the rheology of erythrocytes, the renin-angiotensin system activity and vascular tone [15,16,24]. In the glomerulus, where inflammatory cytokines evoke leukocyte migration, T cell activation, fibrosis, sclerosis and scarring, the value of taurine as an antioxidant is paramount. Taurine scavenges ROS that can influence podocyte function and increase protein excretion. In the proximal tubule, the site of bulk reabsorption of ions, organic solutes and water, taurine influences sodium transport and is taken up itself to maintain the body pool size in an adaptive response to variations in dietary availability. The taurine transporter system maintains the steep plasma (extracellular, μM) to intracellular (mM) concentration gradient despite huge variations in taurine intake. In the medulla, taurine is critical to cell volume regulation, moving into or out of collecting duct cells relative to external osmolarity. Taurine’s role as an osmolyte is likely important in many cell types in nearly all organs, but it is especially evident in renal medullary cells, where final urine concentration is established.

## Abbreviations

AKI: acute kidney injury; DOCA: deoxycorticosterone acetate; FSGS: focal segmental glomerulosclerosis; GABA: gamma-aminobutyric acid; GAP: glomerular albumin permeability; GLUT1: glucose transporter 1; HOCl: hypochlorous acid; HTD: high taurine diet; L-NAME: L-nitro-arginine methyl ester; LTD: low taurine diet; MPO: myeloperoxidase; NO: nitric oxide; NTD: normal taurine diet; PKC: protein kinase C; PAF: platelet-activating factor; RAS: renin-angiotensin system; ROS: reactive oxygen species; TonE: tonicity response element; TonEBP: TonE binding protein; TREE: taurine response element; TNF: tumor necrosis factor ; UW: University of Wisconsin

## Competing interests

The authors declare that they have no competing interests.
